# Quantifying Stratospheric Temperature Signals and Climate Imprints From Post‐2000 Volcanic Eruptions

**DOI:** 10.1029/2019GL084396

**Published:** 2019-11-03

**Authors:** Matthias Stocker, Florian Ladstädter, Hallgeir Wilhelmsen, Andrea K. Steiner

**Affiliations:** ^1^ Wegener Center for Climate and Global Change (WEGC) University of Graz Graz Austria; ^2^ Institute for Geophysics, Astrophysics, and Meteorology/Institute of Physics University of Graz Graz Austria; ^3^ FWF‐DK Climate Change University of Graz Graz Austria

**Keywords:** volcanic signals, stratospheric temperature, temperature trends, satellite observations

## Abstract

Small volcanic eruptions and their effects have recently come into research focus. While large eruptions are known to strongly affect stratospheric temperature, the impacts of smaller eruptions are hard to quantify because their signals are masked by natural variability. Here, we quantify the temperature signals from small volcanic eruptions between 2002 and 2016 using new vertically resolved aerosol data and precise temperature observations from radio occultation. We find characteristic space‐time signals that can be associated with specific eruptions. In the lower stratosphere, robust warming signals are observed, while in the midstratosphere also cooling signals of some eruptions appear. We find that the volcanic contribution to the temperature trend is up to 20%, depending on latitude and altitude. We conclude that detailed knowledge of the vertical structure of volcanic temperature impacts is crucial for comprehensive trend analysis in order to separate natural from anthropogenic temperature changes.

## Key Points

- The imprints of minor volcanic eruptions on stratospheric temperature are precisely quantified
- Vertically high resolved aerosol and temperature observations facilitate the accurate detection of volcanic signals in space and time
- Minor volcanic eruptions clearly affect the short‐term temperature trend in the lower stratosphere

## 1 Introduction

Volcanic eruptions can substantially influence the climate system (Kremser et al., 2016; Robock, 2000, 2015; Timmreck, 2012) through the emission of trace gases, volcanic ash, and aerosol forming substances, such as sulfur dioxide (SO_2_). In the troposphere, the volcanic ash causes local weather changes (Robock, 2015). In the stratosphere, sulfate aerosols formed by volcanic SO_2_ emissions absorb and backscatter solar and terrestrial radiation and also enhance chemical reactions, for example, ozone depletion. This affects surface temperature as well as stratospheric temperature and dynamics (Aquila et al., 2013; Robock, 2000, 2015). Those sulfate aerosols are among the main components of the stratospheric aerosol layer (Kremser et al., 2016). Besides aerosol chemistry and physics, their distribution in the stratosphere is governed by large‐scale processes such as the Brewer Dobson Circulation and the Quasi‐biennial Oscillation (QBO; Baldwin et al., 2001; Kremser et al., 2016; Timmreck, 2012; Trepte & Hitchman, 1992).

The last major volcanic eruptions were those of El Chichón in 1982 and Pinatubo in 1991. They emitted tremendous amounts of SO_2_, and their consequences have been addressed in several studies (e.g., Aquila et al., 2013; Free & Lanzante, 2009; Randel et al., 2009). They were found to strongly affect tropospheric as well as stratospheric temperature and chemistry. Following the Pinatubo eruption, a global tropospheric cooling of up to 0.6 K (Parker et al., 1996) and a stratospheric warming of 2 K (Robock, 2000) were detected. In the lowermost stratosphere, model studies showed an even stronger warming due to the Pinatubo aerosols compared to the observed one (Free & Lanzante, 2009). Moreover, Aquila et al. (2013) modeled a drop of approximately 8% in tropical stratospheric ozone concentration directly after the eruption.

Recently, a series of smaller volcanic eruptions that started in 2002 has been of research interest. Those eruptions most likely led to a steady increase in stratospheric aerosol optical depth (AOD) during the last decade (Mehta et al., 2015; Vernier et al., 2011). Also, a contribution of those eruptions to the 21st century warming hiatus has been discussed (Santer et al., 2015).

Not all of the small eruptions reach the stratosphere, depending on the latitude of the eruption, the explosivity, the emitted SO_2_ mass (Robock, 2000), and also the emission height. Only aerosols reaching the stratosphere have a sufficient lifespan (up to 3 years) to affect climate (Robock, 2015).

The Volcanic Explosivity Index (VEI), which describes the magnitude of the eruption, is a first indication of whether the eruption can cause stratospheric temperature changes. For post‐2000 volcanoes, eruptions that reached the stratosphere typically had a minimum VEI of 4 (Table 1). Yet, not all of the >VEI 4 eruptions reached the stratosphere. The Puyehue in 2011, for example, had a VEI of 5, but most of the emitted aerosols were not transported to altitudes above 14 km. Data for these indicators used here are taken from the Global Volcanism Program (2013) database, “Volcanoes of the World.”

**Table 1:** Eruptions Between 2002 and 2017 With a Minimum VEI of 4

Name	Start date	VEI	SO_2_ mass (kt)	SO_2_ altitude (max.; km)	Latitude	Country
**Ruang (Ru)**	09‐25‐2002	4	80	22	2.3°N	Indonesia
Reventador	11‐03‐2002	4	84	17	0.077°S	Ecuador
**Manam (Ma)**	10‐24‐2004	4	152^a^	24^a^	4.08°S	Papua New Guinea
**Rabaul (Tavurvur; Ta)**	08‐11‐2006	4	300	18	4.271°S	Papua New Guinea
Chaitén	05‐02‐2008	4	14	17	42.833°S	Chile
Okmok	07‐12‐2008	4	150	15	53.43°N	United States (Alaska)
**Kasatochi (Ka)**	08‐07‐2008	4	2,000	15	52.177°N	United States (Alaska)
**Sarychev Peak (Sp)**	06‐11‐2009	4	1,200	17	48.092°N	Russia
Eyjafjallajökull	03‐20‐2010	4	466	9	63.633°N	Iceland
**Merapi (Me)**	10‐26‐2010	4	300	17	7.54°S	Indonesia
Grímsvötn	05‐21‐2011	4	300	12	64.416°N	Iceland
Puyehue‐Cordón Caulle	06‐04‐2011	5	200	14	40.59°S	Chile
**Nabro (Na)**	06‐13‐2011	4	3,650	18	13.37°N	Eritrea
Tolbachik	11‐27‐2012	4	200	10	55.832°N	Russia
Sinabung	09‐15‐2013	4	20	7	3.17°N	Indonesia
**Kelut (Ke)**	02‐13‐2014	4	200	19	7.93°S	Indonesia
**Calbuco (Ca)**	04‐22‐2015	4	400	20	41.33°S	Chile
Wolf	05‐25‐2015	4	200	7	0.02°N	Ecuador

*Note*. Eruptions reaching the stratosphere are in bold. Data are from the Global Volcanism Program (2013).

a Main emission event during the eruption.

The impact of specific smaller eruptions on atmospheric temperature has been addressed by Wang et al. (2009), who investigated the Mount Chaitén eruption in 2008, and Okazaki and Heki (2012), who studied the Eyjafjallajökull eruption in 2010 and the Puyehue eruption in 2011. They found significant negative tropospheric temperature signals induced by those eruptions using novel radio occultation (RO) satellite data. Biondi et al. (2017) also employed RO data to observe the volcanic clouds of the Puyehue and the Nabro eruptions in 2011. For Puyehue they found tropospheric cooling signals, while for Nabro considerable stratospheric warming was detected. Mehta et al. (2015) examined eruptions between 2001 and 2010 using RO and found small but significant warming in the upper troposphere‐lower stratosphere region for the Soufrière Hills and Tavurvur eruptions, which occurred in quick succession.

However, the accurate quantification of temperature signals of such small eruptions is challenging. The emitted SO_2_ mass of these eruptions (Table 1) is up to three orders of magnitude smaller than that of, for example, Pinatubo (20 Mt), illustrating the difficulty. Previous studies relied on AOD data as a proxy for the identification of volcanically induced temperature signals. Yet the AOD does not account for a vertically resolved distribution of the volcanic aerosols, which are lofted within the “tropical pipe” (Flury et al., 2013; Kremser et al., 2016; Vernier et al., 2011).

In this study, we quantify the stratospheric volcanic temperature signals in space and time for post‐2000 volcanoes. We use the potential of a newly available, vertically resolved aerosol data set in combination with precise temperature observations from RO. We focus on the tropical stratospheric region and accurately characterize natural atmospheric variability to investigate the imprint of volcanic signals on short‐term temperature trends.

## 2 Data and Method

### 2.1 Aerosol and Temperature Data

**Figure 1:**
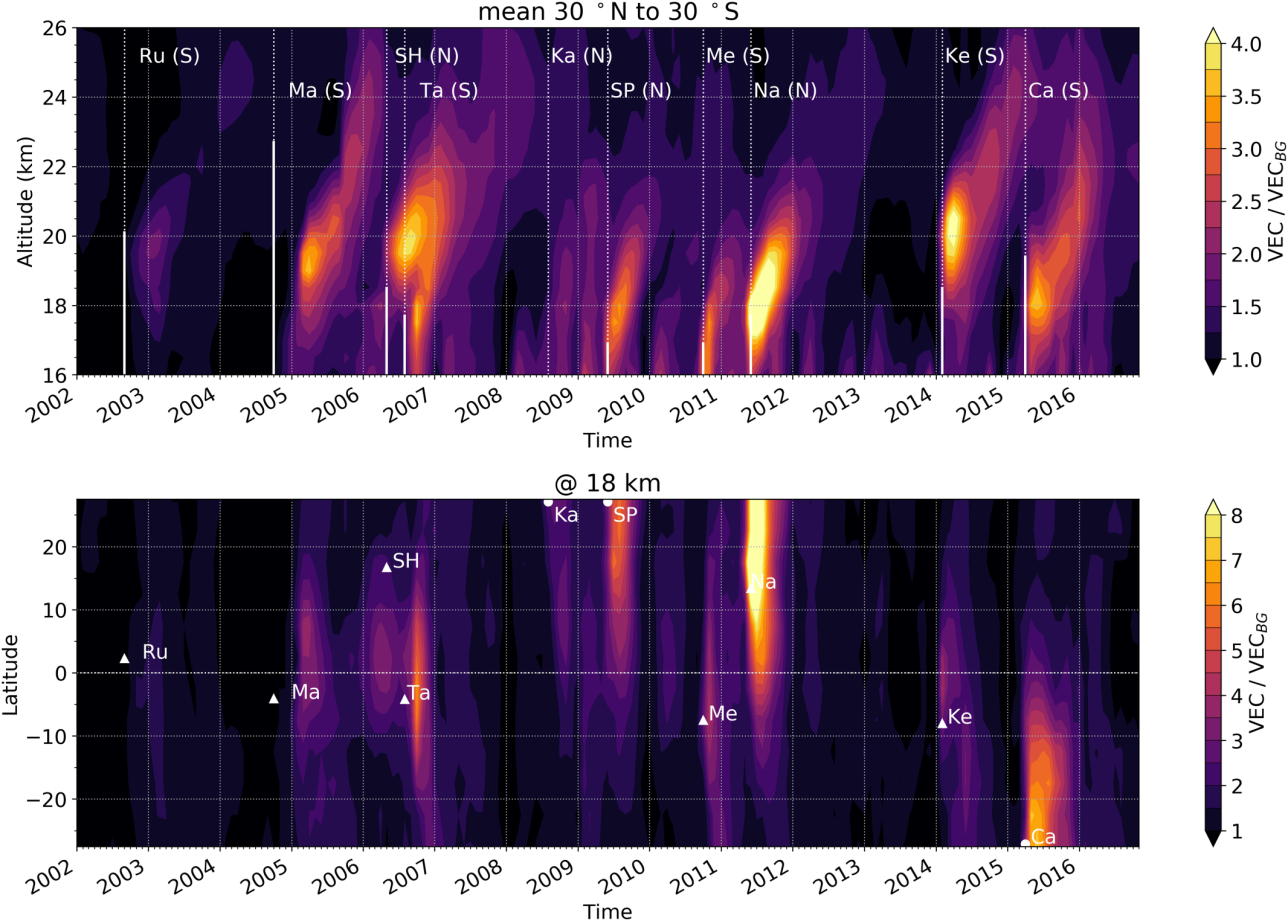
Altitude time pattern of the aerosol concentration divided by the background concentration (mean 30°N to 30°S; top) as well as the latitude time pattern of the aerosol concentration divided by the background concentration at 18 km (bottom). Vertical lines in the altitude time pattern mark the date of the eruptions as well as the maximum SO_2_ altitude reported in the Global Volcanism Program (2013) database (solid lines). N and S in brackets indicate north and south of the equator. Triangles in the latitude time pattern mark the time of the eruption as well as the latitude of the eruption. Eruptions that occurred at latitudes not part of the plot range are marked with a semicircle. Note that, for example, for the Manam (Ma), the start date of the eruption does not coincide with the date of the main emission event.

We use stratospheric aerosol data from the Global Space‐based Stratospheric Aerosol Climatology (GloSSAC; Thomason, 2017; Thomason et al., 2018). GloSSAC is based on various aerosol measurement missions (e.g., SAGE, OSIRIS and CALIPSO; Thomason et al., 2018) and became available in 2017. We employ the vertically resolved Volume Extinction Coefficient measured at 525 nm with a latitudinal resolution of 5° zonal bands and a vertical resolution of 500 m. We inspect altitudes above 16 km as below this level, tropical data are not complete. Since the focus is on the tropical upper troposphere‐lower stratosphere region, the upper limit is set to 26 km and we use data between 30°S to 30°N. From the original aerosol concentration data, we subtract the provided background aerosol concentration to create deseasonalized aerosol anomalies. The background aerosol concentration represents the state of the stratosphere without volcanic influence and is the monthly average concentration of the years 1999 to 2004, excluding 2002 since in this year the eruption of the Ruang occurred (Thomason, 2017; Thomason et al., 2018). The aerosol concentration divided by the background concentration is illustrated in Figure 1 and clearly shows a volcanic pattern of aerosols which are lofted into the middle stratosphere over time.

We take advantage of the vertically high resolved data from RO to analyze the spatiotemporal imprint of volcanoes on temperature. RO is a limb sounding technique exploiting signals from Global Navigation Satellite Systems and provides long‐term stability, global coverage, high accuracy, and vertical resolution in the upper troposphere and lower stratosphere (Steiner et al., 2011). We use temperature data from the Wegener Center OPSv5.6 RO multisatellite record (Angerer et al., 2017) on 5° latitudinal bands with a vertical gridding of 500 m to match the aerosol data. Temperature anomalies are created by subtracting the mean seasonal cycle for the investigated time series.

The GloSSAC data set is currently available from 1979 until the end of 2016. The RO temperature record is available from end of 2001 onward. Therefore, our investigated time period is from 2002 to 2016.

### 2.2 Regression Analysis

We estimate the volcanic signals in stratospheric temperature using multiple linear regression analysis. To account for autocorrelation in the monthly temperature data, we utilize a Generalized Least Squares with Autocorrelated AR(p) Errors model.

For the small eruptions in the study period, temperature signals are hard to detect because they are masked by natural variability. For large eruptions, volcanic signals can be estimated from the regression residuals when natural variability modes such as El Niño–Southern Oscillation (ENSO) or QBO are taken into account. Conventional variability indices, such as the Niño 3.4 SST or QBO winds at specific pressure levels, have been used in previous studies (Randel et al., 2009). This approach is less effective for smaller eruptions, because the residual variability is comparable to the volcanic signal amplitude (Mehta et al., 2015).

To separate the small post‐2000 volcanic signals from other variability, we therefore take advantage of the temperature variability indices introduced by Wilhelmsen et al. (2018), which leave very small temperature residuals by construction. Compared to conventional indices these indices are of high vertical resolution and are created directly from the temperature anomalies derived from the RO measurements, using an empirical orthogonal function (EOF) analysis (Hannachi et al., 2007) on each separate altitude level in the data set. The leading principal components (PCs) are used as variability indices for the corresponding altitude level.

These variability indices do not only include QBO or ENSO, but all other main modes of variability such as aerosol‐induced temperature changes, which leads to a nonzero correlation between the aerosol index and the variability indices. Additionally, the aerosol distribution itself is correlated to the QBO phase (Trepte & Hitchman, 1992). This means that a regression analysis, using only the regular aerosol anomalies as explanatory variable, would result in volcanic temperature signals that also include QBO related temperature variability. Therefore, when both the aerosol and the temperature variability indices are used in the multiple linear regression analysis, this results in collinearity between the regressors (see Santer et al., 2001).

To avoid collinearity, we remove in a first step the QBO signal from the aerosol index, and in a second step the resulting QBO‐free aerosol signal from the variability indices.

In the first step, we subtract the QBO signal from the aerosol anomalies using linear regression. Hereby, we utilize QBO indices derived from the Singapore wind fields via EOF analysis. The reconstructed aerosol concentration for the leading two PCs derived from the wind field indicates a relatively weak QBO signal in the lower stratospheric aerosol concentration, which becomes stronger toward the middle stratosphere. Around 24 km, the impact of the QBO phases on the aerosol anomalies is reversed, changing from positive to negative, and vice versa. This agrees with the QBO‐induced aerosol mixing ratio anomalies found by Hommel et al. (2015), who investigated the influence of the QBO on stratospheric aerosol distribution using an aerosol‐coupled climate model.

After subtracting the QBO reconstructed aerosol concentration from the aerosol anomalies, primarily the volcanic pattern remains in the aerosol regressor, as verified by correlation analysis.

In the second step, we create the temperature variability indices from the residual temperature anomalies that remain after excluding the aerosol variability. This is done by subtracting a first estimate of the volcano‐related temperature variability from the original temperature anomalies. The first estimate is computed using the Generalized Least Squares with Autocorrelated AR(p) Errors model on the temperature anomalies with the volcanic aerosols distribution, as the only explanatory variable. Since we removed the QBO signals from the aerosols distribution the first estimate of the volcanic temperature signals does not include QBO variability. Then we apply an EOF analysis on the residual temperature anomalies as explained above, and use the resulting variability indices in the following steps.

The final regression model then includes the QBO‐free aerosol anomalies as well as the aerosol‐free variability indices derived from the temperature anomalies after subtracting the estimated volcanic temperature signal. It can be written as

(1) $$X_{\phi ,h,t}^{\text{Mod}}=\beta_{\phi ,h}^{\text{const}}+\beta_{\phi ,h}^{\text{linear}}t+\beta_{\phi ,h}^{\text{aer}}A_{\phi ,h,t}^{\text{anom}}+\beta_{\phi ,h}^{\text{PC1}}{\text{PC1}}_{h,t}+\beta_{\phi ,h}^{\text{PC2}}{\text{PC2}}_{h,t}+\beta_{\phi ,h}^{\text{PC3}}{\text{PC3}}_{h,t}+\epsilon$$

where X ${}_{\phi ,h,t}^{\text{Mod}}$ represents the temperature anomalies as function of latitude (*ϕ*), altitude (*h*), and time (*t*), A ${}_{\phi ,h,t}^{\text{anom}}$ the QBO‐free aerosol anomalies, PC1_*h*,*t*_, PC2_*h*,*t*_, and PC3_*h*,*t*_ the aerosol‐free variability indices, and *ϵ* the residual.

## 3 Results and Discussion

Signals from several central tropical eruptions such as those of Manam, Soufrière Hills and Tavurvur, Merapi, Nabro, and Kelut are clearly visible in the aerosol data (Figure 1). Yet, also perturbations from higher‐latitude eruptions such as those of Sarychev Peak and Calbuco can be identified. After the eruptions, the aerosols rise with roughly 4 km/year (cf. Vernier et al., 2011) in the lower to middle tropical stratosphere due to the vertical transport within the tropical pipe. However, a considerable amount of aerosols reaches higher altitudes only after particular eruptions such as that of Tavurvur and Soufrière Hills, respectively and also of Kelut and Calbuco. From Nabro, which emitted several times more aerosols than the other eruptions, seemingly only a small amount reached altitudes above 20 km.

This difference in the efficiency of the vertical transport of the aerosols not only depends on the injection height but also on the phase of the QBO during the time of the eruption, since the strength of the horizontal and vertical transport processes within the tropical pipe are connected to the phase of the QBO (Flury et al., 2013; Kremser et al., 2016). Flury et al. (2013) note that in the westerly shear zone of the QBO the transport out of the tropical pipe toward higher latitudes is enhanced while the vertical transport within the pipe is reduced. This means that during the QBO westerly shear volcanic aerosols are less likely transported to higher altitudes but are rather transported to the midlatitudes. Conversely, during the QBO easterly shear higher altitudes are more likely to be reached.

### 3.1 Volcanic Temperature Signals

**Figure 2:**
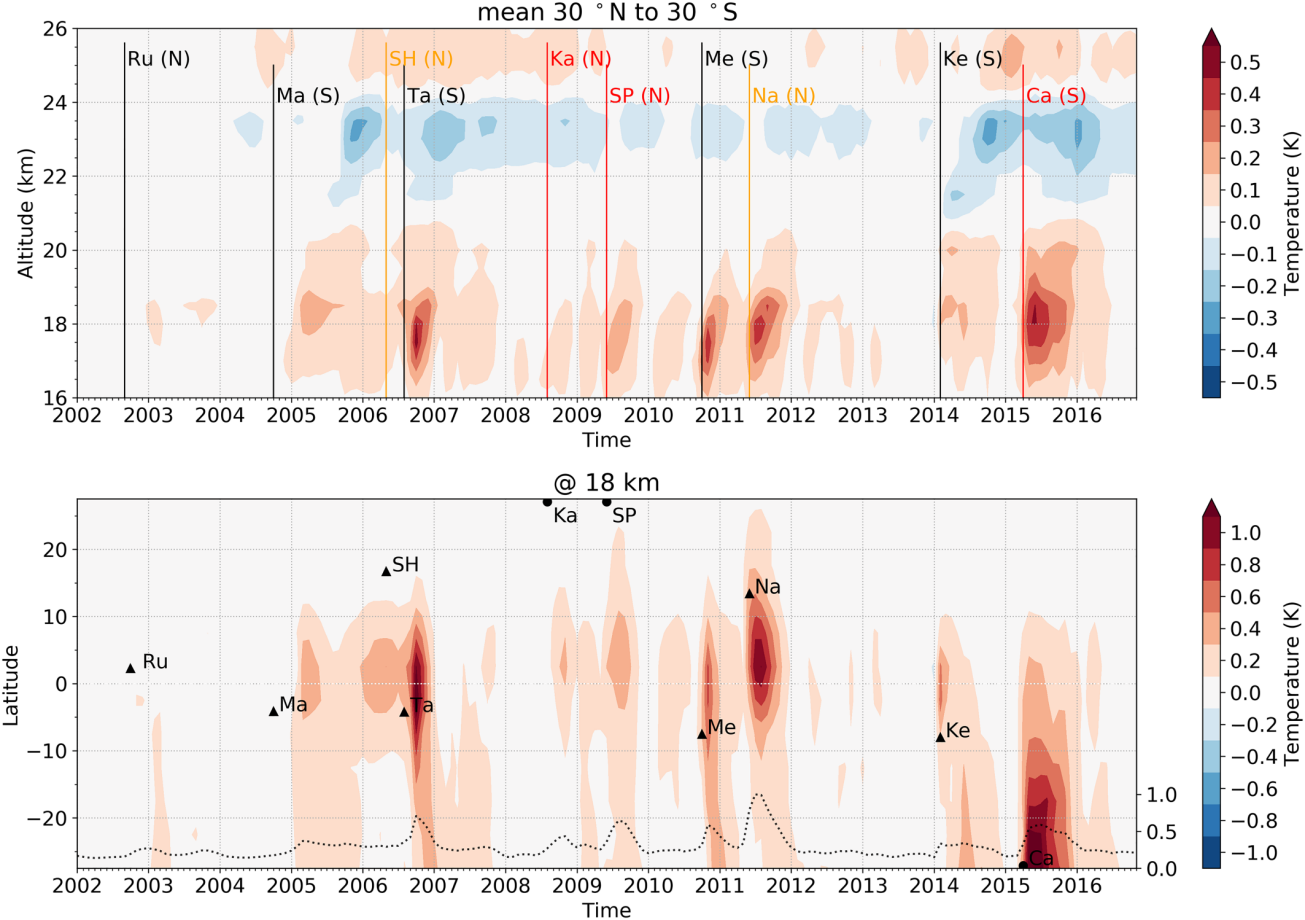
Altitude time cross section of the volcanic aerosol reconstructed temperature (mean 30°N to 30°S; top) as well as the latitude time cross section at 18 km (bottom). Vertical lines in the altitude time pattern mark the date of the eruptions. N and S in brackets indicate north and south of the equator. Line colors indicate the latitude of the eruption (red >30, orange >10, and black <10). Triangles in the latitude time pattern mark the date as well as the latitude of the eruption. Eruptions that occurred at latitudes not part of the plot range are marked with a semicircle. The dashed line represents the normalized VEC for the 18‐km altitude level (mean 30°N to 30°S).

In the reconstructed temperature for the volcanic aerosols (Figure 2), warming signals following the different eruptions are visible in the lowermost stratosphere up to around 20 km. Especially the Tavurvur (2006), Merapi (2010), Nabro (2011), and Calbuco (2015) eruptions show strong temperature impacts of about 0.5 K in the tropical mean. Weaker, but also visible signals of about 0.2 K can be associated with the Manam (2005), the Sarychev Peak (2009), and the Kelut (2014) eruptions. The warming at this altitude is most likely a result of absorption of solar and terrestrial long wave radiation by the volcanic sulfate aerosols (Robock, 2015, 2000; Mehta et al., 2015). The magnitude of the temperature increase following the Tavurvur and Soufrière Hills eruptions is consistent with the warming signal found by Mehta et al. (2015), who estimated the warming signal as the difference of averaged temperature residuals before and after the eruptions. Nabro emitted far more aerosols to this altitude, the subsequent warming, however, is only slightly stronger than for the Soufrière Hills and Tavurvur eruptions. This results from the Nabro aerosols being spread predominantly to the Northern hemisphere by the Asian monsoon (Bourassa et al., 2012). Thus, the main aerosol concentration is located at latitudes north of 10°N (Figure 1, bottom). For the Calbuco eruption, in contrast, a large amount of aerosols was transported toward the equator.

The altitude time cross section (Figure 2, top) further displays cooling signals of up to 0.3 K between 20 and 24 km. Since the signals follow the pattern of the volcanic aerosols as seen in Figure 1, we assume that they originate from the specific eruptions, which affected the aerosol concentration at altitudes between 20 and 24 km. Eruptions that have an impact on temperature at this height are those of Manam (2005) and Tavurvur and Soufrière Hills (2006; cf. Mehta et al., 2015), as well as Kelut (2014) and Calbuco (2015). Other eruptions such as Nabro (2011), which also caused a substantial temperature increase in the lower stratosphere (Biondi et al., 2017), appear to have nearly no cooling effect at higher altitudes. This correlates with the findings in Figure 1 (top), which indicates that the Merapi or the Nabro eruption did not substantially influence the aerosol concentration at altitudes above 20 km.

However, the cooling between 22 and 24 km is an interesting feature that cannot simply be explained by the radiative properties of the sulfate aerosols. A possible explanation for the cooling is given by Robock (2015) and Aquila et al. (2013). As described by Robock (2015), aerosols, in addition to their effects on the radiative flux, can also affect stratospheric dynamics and chemistry (especially ozone depletion). Robock (2015) also notes that in the tropics volcanic clouds enhance the tropical upwelling and hence bring low ozone concentration layers to higher altitudes, possibly causing cooling. Simulations by Aquila et al. (2013) showed a decrease in ozone shortly after the Pinatubo eruption, strongest at about 24 km, which was mainly due to increased tropical upwelling. For large eruptions, the warming effect due to the huge amount of aerosols is dominant (Free & Lanzante, 2009; Randel et al., 2009; Robock, 2015). However, for the small eruptions considered in this study, the dynamical ozone reduction could be more relevant and could explain the observed cooling signals.

A strong indication that the cooling signals are caused by changes in the ozone concentration is that they disappear when we include ozone variability (not shown). The warming signals in the lowermost stratosphere, however, remain robust. As the cooling is assumed to be an aerosol‐induced effect, we do not further account for ozone.

Other possible explanations for cooling signals in the tropical stratosphere are ENSO‐related changes in the ozone concentration due to increased tropical upwelling (Diallo et al., 2018; Domeisen et al., 2019; Randel et al., 2009). Diallo et al. (2018) found a strong reduction of tropical ozone related to the 2015/2016 ENSO event. We investigated the influence of ENSO on the volcano‐induced temperature variability and found that it is negligible (consistent with Santer et al., 2015). Additionally, the midstratospheric cooling signals in 2010 and 2014 do not coincide with ENSO events. Therefore, we assume that the cooling signals resulting from our approach are not related to an ENSO pattern in the stratospheric circulation.

The small positive temperature signals around 26 km presumably result from volcanic modulations of the aerosol concentration in the stratospheric aerosol layer, which has its strongest mixing ratio at this altitude (Hommel et al., 2015; Vernier et al., 2011).

Figure 2 (bottom) shows the latitudinal distribution of the warming signals maxima at 18 km, which reveals additional features. Except for the Calbuco, the warming signals appear to be limited to approximately 15°N to 15°S in latitude and are largest close to the equator. This even applies to eruptions that took place at high latitudes and of which only a small fraction of aerosols reached the tropical region, where their effect is more pronounced (Ferraro et al., 2011; Mehta et al., 2015).

In contrast to other higher‐latitude eruptions, for the Calbuco eruption in the Southern Hemisphere, a strong warming is observed. As shown in Figure 1 (bottom), a large amount of the Calbuco aerosols was transported toward low latitudes. However, the latitude time cross section in Figure 2 (bottom) also shows that the Calbuco warming for this altitude level is largest outside the central tropics. This may be explained by the fact that at the time of the Calbuco eruption there was an extraordinary strong ENSO event (Stockdale et al., 2017). ENSO events cause a comparatively warm troposphere and also lead to reduced temperature in the lower stratosphere, not only in the tropics but also at midlatitudes (Free & Seidel, 2009). Such conditions increase the warming potential of the volcanic aerosols (Ferraro et al., 2011) and hence may explain why the Calbuco signal is strongest at midlatitudes.

### 3.2 Linear Trend Analysis

**Figure 3:**
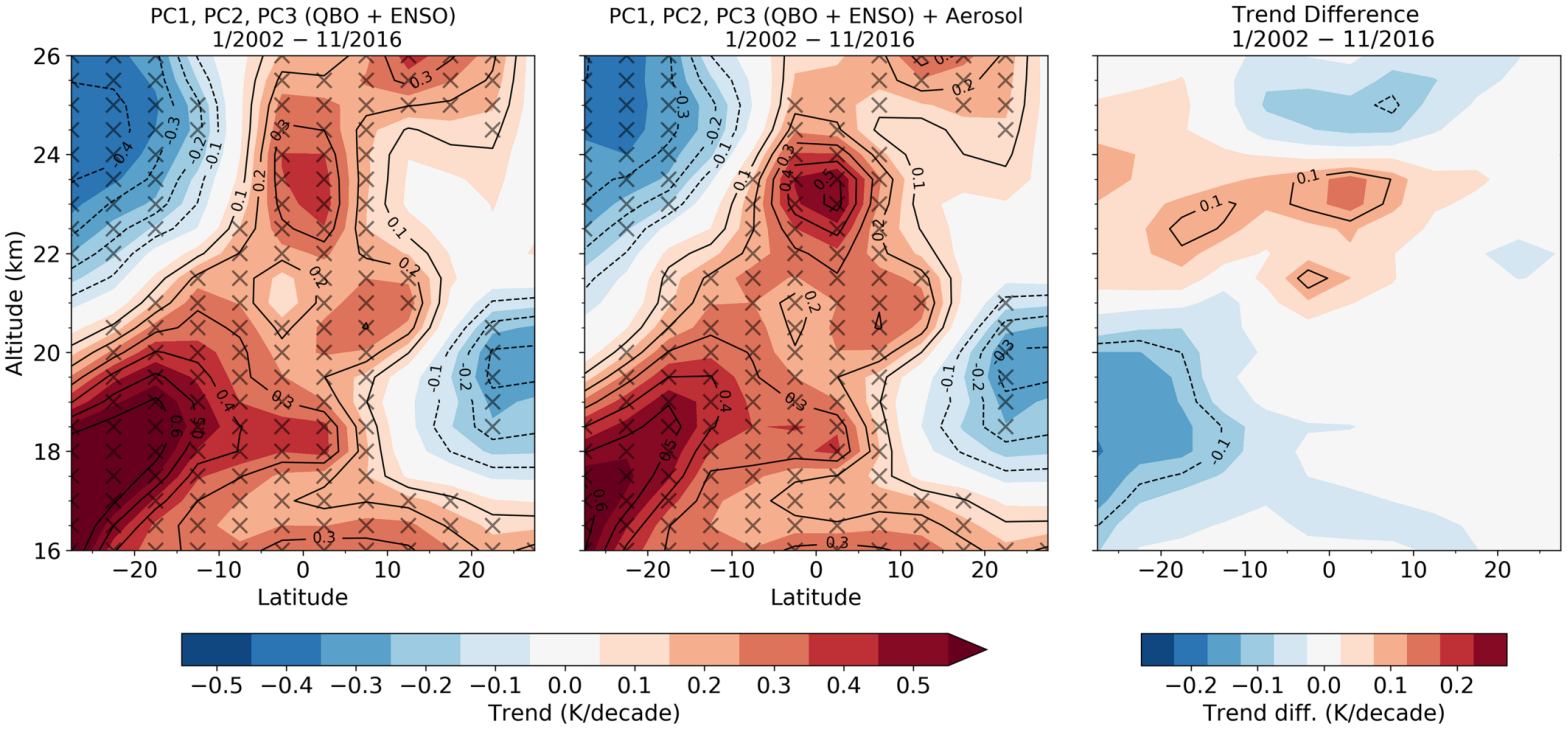
Altitude‐latitude cross section of the linear trend for the time series from 2002 to 2016 considering only natural variability indices (left), and natural variability indices together with the volcanic aerosols (center); trend difference (right). Trend values that are significant at the 95% confidence level are indicated with an X mark. QBO = Quasi‐biennial Oscillation; ENSO = El Niño–Southern Oscillation; PC = principal component.

For analyzing the volcanic impacts on short‐term climatological trends, we perform the regression with and without including aerosols. We find that including the aerosol index causes substantial changes in the resulting linear trend (Figure 3). The differences are more pronounced in the Southern Hemisphere between 30°S and 10°S where the trend is reduced by more than 0.1 K (approximately 20%). This is related to the Calbuco eruption, which occurred at the end of the investigated time series. A slightly increased trend is detected in the equatorial region between 22 and 24 km, presumably due to the cooling signals from the ozone reduction. In the central tropical lower stratosphere, where most of the eruptions show the strongest warming signals, the trend differences are small. This is because the eruptions with strong signals are distributed roughly uniformly in time over the investigated time period.

## 4 Conclusions

This is the first study addressing the precise quantification of the impact of recent minor volcanic eruptions on temperature variability and trends using a combination of vertically high resolved temperature and aerosol data, both from satellite measurements. This facilitates the accurate detection of signals of post‐2000 volcanic eruptions in space and time and their imprint on stratospheric temperature.

We found robust warming signals in the lower stratosphere up to 20 km from the inspected explosive volcanoes. The strongest imprint was found for Tavurvur, Merapi, Nabro, and Calbuco. The latitudinal distribution of the volcanic imprints was clearly resolved and strongest in the central tropics, with the exception of Calbuco. The results for specific eruptions agree with findings of previous studies that used standard AOD to represent volcanic variability.

In the middle stratosphere we found small cooling signals for the investigated volcanic eruptions. A suggested explanation is an indirect aerosol effect on ozone due to an enhanced upwelling of ozone‐poor air after the eruption. The results indicate that small eruptions may also be relevant when investigating stratospheric ozone in the tropics.

Compared to major variability modes such as the QBO, the overall variability due to post‐2000 volcanic eruptions was found to be small; however, they are of importance for short‐term trend analysis. For the investigated time series we found that the impact on linear trends can be up to 20%, depending on altitude and latitude. While the temperature trend is reduced in the lower stratosphere, an enhanced positive trend is observed in the middle stratosphere.

The results show that detailed knowledge of the vertical structure of volcanic temperature changes is crucial for comprehensive trend analysis, as their influence varies for different altitudes and latitudes. Exploiting the potential of the newly available, highly resolved data sets is beneficial for gaining better knowledge on the impacts of volcanic eruptions. This further helps to separate natural climate variability from anthropogenic influences in climate trend detection, and to improve climate models.
